# Badger *Meles meles* as Ecosystem Engineer and Its Legal Status in Europe

**DOI:** 10.3390/ani12070898

**Published:** 2022-03-31

**Authors:** Przemysław Kurek, Łukasz Piechnik, Blanka Wiatrowska, Agnieszka Ważna, Krzysztof Nowakowski, Xosé Pardavila, Jan Cichocki, Barbara Seget

**Affiliations:** 1 Department of Plant Ecology and Environmental Protection, Adam Mickiewicz University, Uniwersytetu Poznańskiego 6, 61-614 Poznań, Poland; 2 Functional and Evolutionary Ecology Group, W. Szafer Institute of Botany, Polish Academy of Sciences, Lubicz 46, 31-512 Cracow, Poland; l.piechnik@botany.pl (Ł.P.); b.seget@botany.pl (B.S.); 3 Department of Forest Botany, Poznań University of Life Sciences, Wojska Polskiego 71D, 60-625 Poznań, Poland; bwiatrowska@interia.pl; 4 Department of Zoology, Institute of Biological Sciences, University of Zielona Góra, Szafrana 1, 65-516 Zielona Góra, Poland; a.wazna@wnb.uz.zgora.pl (A.W.); nowakowski.borsuk@gmail.com (K.N.); j.cichocki@wnb.uz.zgora.pl (J.C.); 5 Sorex Ecoloxia e Medio Ambiente S.L., Rúa das Barreiras 80/2, 15702 Santiago de Compostela, Spain; xosepardavila@yahoo.es

**Keywords:** European badger, Mustelidae, ecosystem engineer, habitat creation, ecosystem services, seed dispersal, hunting season, species diversity

## Abstract

**Simple Summary:**

The European badger, listed in Bern Convention (protected fauna species), is also known as an important ecosystem engineer that creates new microhabitats among its setts suitable for the establishment of many plant and animal species. Badger setts are areas with topsoil disturbance and hence are places with higher species diversity than undisturbed surroundings. Much new data concerning the importance of badgers in ecosystem engineering indicates that the way badger populations are managed in Europe should be reconsidered. Thus, the aim of this study was to review the impact of badgers on shaping of species diversity in forest ecosystems with special attention to their legal status across Europe. The badger is hunted on 69.3% of the continent for 1.5–12 months per year. The real problem with management of this species concerns a quarter of its range in Europe, where the species can be hunted in winter and spring during mating season and during parturition. Reducing the hunting season to a maximum of three months in late autumn (September to November) seems to be a better approach. To prevent the negative effects of overhunting, special attention should be paid to population monitoring.

**Abstract:**

The European badger plays an important role as a natural factor shaping species diversity in forests. Its extensive setts can be used by many other animals as shelters. Soil perturbations in their setts support plant communities that differ from the matrix landscape. The badger is also an effective seed disperser. We investigated its role as an ecosystem engineer in preserving species diversity and discussed its legal status across Europe. In most European countries (69.3% of the continent), the badger is hunted, sometimes year-round. The hunting season lasting through winter until early spring may have a negative effect on badger populations, especially when cubs are born in February. Although this species is Red Listed in 19 European countries (with categories ranging from LC to EN), the badger is strictly protected by law in 30.7% of its European range. A reduction in badger populations may limit its ecosystem services (seed dispersal, topsoil disturbances, microhabitat creation). Much new data on the importance of badgers in ecosystem engineering has allowed us to reconsider how we manage badger populations.

## 1. Introduction

Natural disturbances driving species diversity in European temperate forests (mostly coniferous monocultures) are limited to the primeval or protected forests and are very rare in managed forests. Small-scale disturbances in managed forests are under strict control so that gaps, uprooted trees, or rotten logs can be largely avoided [1]. As a result, European forests have lost their naturalness and diversity but still have a great potential for rewilding [2]. In a great part of Europe, the diversity of plants and animals demanding natural disturbances in forests can be increased by the activity of keystone species such as burrowing mammals. This guild seems to be one of the few remaining natural disturbance agents, causing changes in plant and animal communities and enhancing diversity in the forest floor [3,4]. The best example is the European badger, *Meles meles*, one of the most widespread medium-sized carnivores on the continent [5], and are known for digging extensive burrows, or setts, which vary considerably in internal structure and use [6], and may cover a large area of the forest floor [7,8].

The European badger is a common and widespread species [9,10]. Badgers play an important part in various interspecific interactions depending on their diet, behaviour, prey-predator interactions, disease dispersal, etc. [5,11,12,13,14]. Badger-human relationships can be quite ambivalent due to some damage, e.g., in farmlands or infrastructure [15,16]. It is not commonly known that badger can also be considered ecosystem engineers, acting as a relevant natural factor in shaping species diversity. Extensive and durable setts may be used by many other animals as safe breeding sites or temporary shelters [17,18,19]. Badgers are also effective seed dispersers of fleshy-fruited plants [20,21]. Small-scale local disturbances caused by such semi–fossorial mammals can substantially influence ecosystem structure and functioning. Perturbations (topsoil perturbations) support plant communities that differ qualitatively and quantitatively from the matrix landscape [3]. Some authors reported that topsoil disturbance by digging in temperate habitats has a positive impact on soil properties [22], and therefore setts, are places of higher diversity of vascular plants [3,23,24,25,26,27], bryophytes [4], oribatid mites [28] and other invertebrates [29,30].

Natural processes (seed dispersal and topsoil mixing) initiated by badgers seem to be strongly limited due to many factors associated with these animals, such as landscape transformation [31,32], road accidents [33,34,35,36], natural causes (e.g., interspecific killing [37], diseases [38], intra– and interspecific competition [39,40]) and hunting [41,42]. Beside road accidents and natural mortality causes, hunting may be a relevant additional factor that reduces the number of badgers and disturbs their population functioning [43]. In general, natural factors are beyond our control, but we can reduce the negative effect of some anthropogenic factors. However, it is impossible to exclude them completely (e.g., collisions, poaching) from the environment. Only hunting can be truly controlled. In Europe, the need to control hunting of badgers is being considered, as reflected in (protected fauna species) of the Bern Convention, where the badger is mentioned. Moreover, much of the new data on the badger as an ecosystem engineer can provide the basis for future reconsideration of its legal status in Europe. The aim of this study was to review the impact of badgers on shaping species diversity in forest ecosystems, with special attention to their legal status in European countries.

## 2. Data Collection

**Figure 1:**
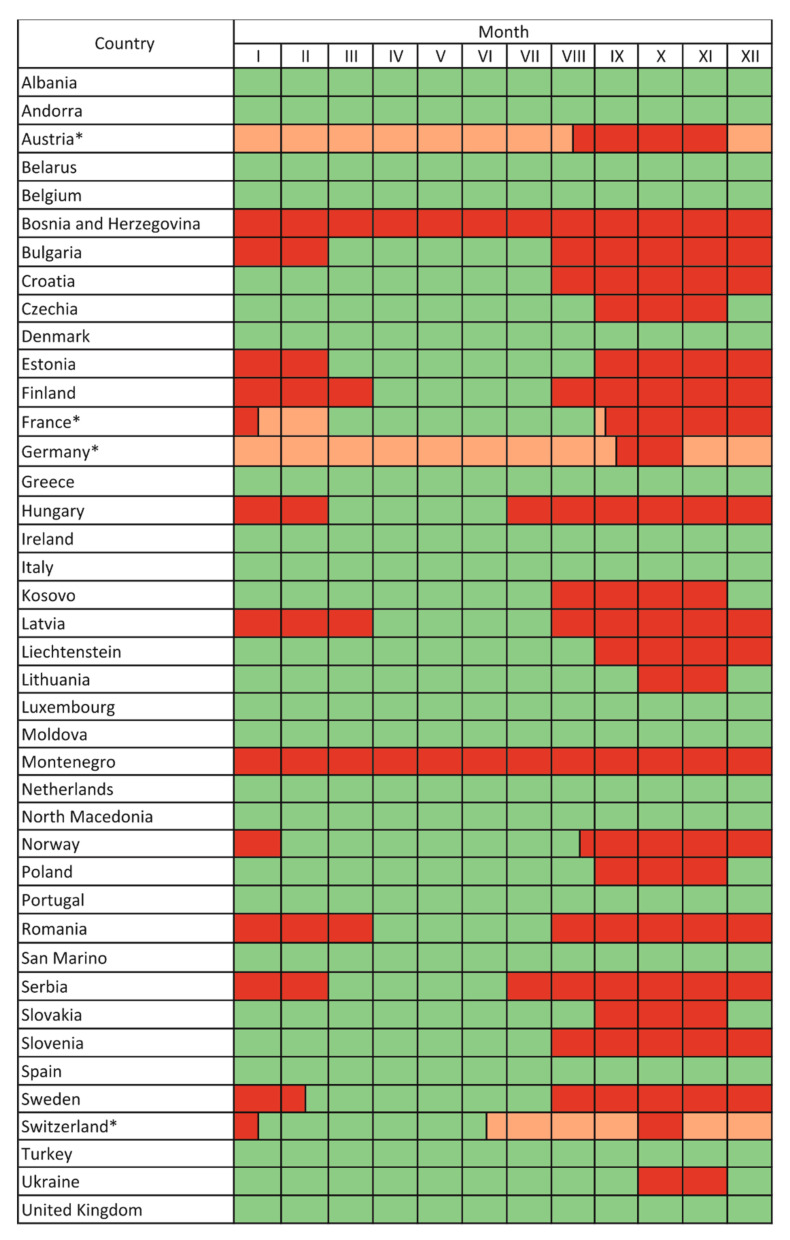
General hunting seasons of the European badger *Meles meles* in European countries. Green area–species protected or hunting suspended; red area–species hunted; *—countries where hunting period differs between regions (for more details, see Table 1); for these states, minimum (red area) and maximum (pink area) hunting seasons are presented.

**Figure 2:**
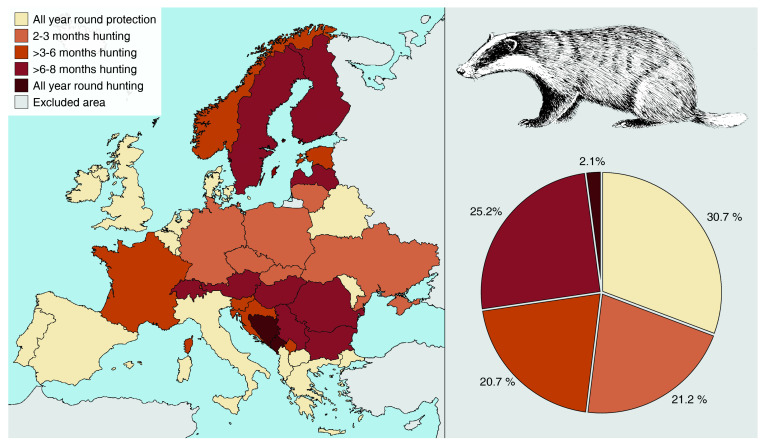
General hunting seasons of European badger *Meles meles* in Europe. The percentages in the pie chart indicate the range share of a given legal status of the badger. Countries where hunting season differs between regions (Austria, France, Germany, and Switzerland) classified as explained in Methods, more detailed data presented in Table 1. Updated to 30 June 2020.

Data on the legal status of the badger in Europe were searched for using Google Scholar (key words: badger, protection, legal status, list of protected species) and websites presenting current law in national languages (sources presented in Table 1). We cited papers, legal documents, websites, and personal correspondence–especially when the law required interpretation and explanation. Countries where badgers are absent (Cyprus, Iceland, Malta) were excluded from consideration (see [44]). The Caucasus, the Asian part of Turkey and the European part of Russia were also excluded from the analysis. Data were gathered until 30 June 2020. On this basis, we distinguished three status categories presented in Table 1: P = badger protected all year round, H = hunted within a hunting season, and U = unclear, where the badger is considered as a game species, not hunted but also not protected. Similarly, other data were collected, such as badger’s presence in a national red book/list and some explanatory information when hunting law was regulated in autonomous regions. As there are no general data on hunting bags in Europe, we gathered them from websites and reports with national statistics published by hunting societies. If no data were found, we contacted wildlife managers and other administrative staff who had access to the data. A similar procedure was followed for the estimation of the hunting season duration. For federations with regionally regulated hunting laws, Figure 1 presents the minimal and maximal hunting season (Austria, France, Germany, Switzerland; see also Table 1). For the analysis presented in the map in Figure 2, countries with hunting season regulated only regionally were classified according to the hunting season that prevails at the state’s area (Austria). Other federal countries (France, Germany, and Switzerland) were classified according to the general hunting season as their overriding federal law stated. For the analysis presenting the percentages of areas in Europe with different hunting seasons (pie chart in Figure 2), more detailed regional data were used for all federal countries (see Table 1).

## 3. European Badger as Ecosystem Engineer

### 3.1. Interaction with Soil and Plants

Badgers interact with other ecosystem components in the following two ways: (1) by spreading seeds [119,120] and (2) inducing changes in chemical and physical properties of the topsoil among setts, thereby creating favourable conditions for colonisation by various plants and animals [3].

The European badger markedly affects physical and chemical soil properties, the local relief of the forest floor, and the species richness of other groups of organisms [3,6,121]. Its impact is due to the long-term habituation of extensive setts, which can cover an area of up to 970 m^2^ [8], where the volume of excavated soil can reach up to 28 m^3^ [121]. This is why old burrows are distinguished by a different surface shape than their surroundings. The soil of mounds is characterized by increased pH (mounds: 5.5; reference areas: 4.3) and higher concentrations of nutrients, including Ca (more than thrice) and Mg (almost twice) in comparison to undisturbed soil [3]. Mounds of dug soil from deeper horizons containing less organic matter have low carbon and nitrogen content. Higher availability of phosphorus has been reported on the mounds, which may be associated with higher pH [3].

Long-term use of setts and permanent topsoil mixing and changes in chemical and physical properties lead to an increase in plant species richness, in comparison to undisturbed surroundings [3,27]. Soil digging by badgers results in the appearance of ruderal plants associated with unstable environments and tolerating disturbances, such as *Urtica dioica* [25]. The presence of this group of plants suggests that the increased species richness within the setts is significantly related to the disturbance and consequent nutrient enrichment of the topsoil. Similar observations apply to bryophytes as follows: on average, 9.5 species were found in the setts, 4.5 of them in undisturbed soil [4]. Moreover, 38.5% of species occurring exclusively on burrows were identified as epiphytic bryophytes on the bark of fleshy-fruited shrubs growing mostly on badger burrows. It means that there is also a secondary effect of diversity shaping by burrowing badgers [4]. Topsoil disturbance plays a vital role in shaping plant diversity and is mainly manifested by the reduction of competition from dominant species and the creation of survival opportunities for young plants. Beneficial changes in soil chemistry (higher pH and nutrients content) are an additional disturbance effect used by plants [44].

In addition, badgers eat fleshy fruits of many plant species, as seeds of at least 13 species were found in the faeces of this carnivore [21]. The role of the badger in seed dispersal is repeatedly emphasized, especially for species producing large fruit, e.g., pears *Pyrus* sp., as its seeds cannot be easily eaten and dispersed by birds [119]. Seeds from fruit eaten by badgers are deposited in latrines, which are located on the territory boundary and in the vicinity of setts. As a result, we can observe a greater species richness of fleshy-fruited trees and shrubs near old burrows that have been exploited for many years [3].

### 3.2. Interactions with Fauna

The badger interacts with other animals in many different ways. As a predator, it naturally interacts with its prey populations [122] and plays an important role in trophic interactions, e.g., in mechanisms of mesopredator release [11]. Sometimes it becomes prey for other carnivores [123]. An interesting aspect of interactions with other mammals is the sharing of burrows, e.g., with the raccoon dog, *Nyctereutes procyonoides*, the red fox, *Vulpes vulpes*, the golden jackal, *Canis aureus*, the crested porcupine, *Hystrix cristata*, or the Egyptian mongoose, *Herpestes ichneumon* [18,19,23,25,124,125,126,127]. Badger setts can also serve as shelter for many other animals. For example, in the Albera Massif in Spain, many of the Greek tortoises *Testudo hermanni,* survived a forest fire hidden in badger setts [128]. Moreover, fire salamander *Salamandra salamandra* in the Polish Carpathians was recorded in badger setts [43]. Little is known about invertebrates that find shelter inside the setts or inhabit the soil within them. Hancox [17] in his review mentioned 89 species of arthropods found in badger burrows, representing beetles Coleoptera (61 species), flies Diptera (18 species), butterflies Lepidoptera (one species—*Noctua pronuba*) and mites Acarina (8 species).

Badgers may gather lots of organic material in chambers when building their nest for winter torpor and rearing cubs. Nest material can exceed a volume of 37 dm^3^ [6]. Such accumulation of organic matter creates ideal conditions for many saprotrophic invertebrates, including mites (Acari). Sixteen species of Uropodina mites were recorded in badger nests [29]. Two of them, *Trematura patavina* and *Nenteria oudemansi*, were represented by all developmental stages there (protonymphs, deutonymphs, and adults of both sexes), in contrast to the Uropodina mite community from nests of other fossorial mammals, e.g., moles, *Talpa europaea* [29]. These results showed that badger nests are potential habitats for many saprobiontic taxa that enhance species and niche diversity in ecosystems.

Soil-dwelling fauna also benefits from topsoil mixing in badger setts, where higher species richness is observed in comparison to intact soils in their surroundings. Oribatida communities differed both qualitatively and quantitatively as follows: 52 species were found in the soil of old burrow mounds, compared to 36 species from reference plots [28]. Moreover, mounds of excavated soil were inhabited by 20 species of oribatid mites, which were not found anywhere else outside the mounds. Thus, badgers create new microhabitats (mounds of excavated soil) that shape species richness and differentiate the communities of soil-dwelling fauna in their setts. Such differences may persist for a long time, even after the hosts have abandoned the setts. Badgers may dig and use more burrows within their territories [129], thus, their impact on forest floor diversity is spatially extensive, not limited only to main setts.

## 4. The Legal Status of Badger across Europe

In over half of the continent, badgers are considered game species, in some cases with hunting seasons of up to eight months per year or no protection period at all (Table 1). The shortest known hunting season of 2–3 months applies in Ukraine, Czechia, Lithuania, Poland, Slovakia and some parts of Germany, covering 21.2% of Europe excluding Russia. A slightly longer hunting season, extending into late autumn and early winter, has been adopted in Croatia, Kosovo and Slovenia. There is also a group of countries where the hunting season is longer, extending to winter and early spring (Figure 1). Hunting in winter may have a negative effect on badger populations, especially when the young are born in February. This is the main argument that badger hunting should be forbidden for most of the year, including periods of parturition and rearing cubs. This applies to a group of countries where the hunting season extends into February or March (Bulgaria, Estonia, Finland, France, Hungary, Latvia, Romania, Serbia) or where it continues throughout the year (Steiermark in Austria, Mecklenburg-Vorpommern and Sachsen In Germanyy, Bosnia and Herzegovina). The legal status of this species varies widely in countries composed of autonomous regions and may depend on the law adopted in particular regions at a particular time. For example, in federal countries (Austria, Bosnia and Herzegovina, Germany, Switzerland, France), the legislation of individual regions takes precedence over federal law and has a different, usually longer, hunting season. As a result of different approaches to badger hunting presented in many European countries, the protection period is very short or may not even exist in some parts of the continent (Figure 1).

According to the IUCN Red List, the European badger is in the LC (least concern) category [130,131]. However, the species is mentioned in Red Data Books/Red Lists of animals in 19 European countries where it is simultaneously protected (Belarus, Denmark, Spain, Italy, Portugal) or hunted (Austria, France, Czechia, Kosovo, Norway, and Slovakia) [49,51,67,79,96,102,107]. Its classification as LC prevails (*n* = 14) in most European countries, but in the following others the threat category is higher: VU in Flanders (Belgium), Murcia (but LC in other parts of Spain), Belarus and Slovakia or even EN in Albania (Table 1).

Badgers are classified as protected species in several countries (Albania, Andorra, Belarus, Denmark, United Kingdom, Ireland, the Netherlands, Belgium, Luxembourg, Spain, and Portugal, Figure 2). An intermediate situation occurs in Italy, Moldova, North Macedonia, and Greece, where its status is unclear, as it is not subject to species protection and, at the same time it is not a game species and, de jure, it cannot be hunted. However, legal protection of the badger may have some exceptions, and this does not always mean that the species is not hunted at all. In Great Britain and Ireland, badger protection can be waived as part of the fight against bTB [132].

Relative to the area of Europe, the species is protected by law (or at least not hunted) in 30.7% of the range analysed in this study (Figure 2). In 27.3% of the area of Europe, hunting is permitted during a very long hunting season of more than half a year (from late summer to late winter) that covers the time when females are just before or during parturition. Badgers are still hunted year-round in at least 2.1% of the study area (Figure 2). In reality, this percentage is even higher as some countries with shorter overall hunting seasons have recognised exceptions to this rule and badger hunting has been allowed all year round, i.e., in regions where capercaillie *Tetrao urogallus*, black grouse *Lyrurus tetrix*, hazel grouse *Tetrastes bonasia* and partridge *Perdix perdix* breed (Poland, Czechia) or close to human settlements and hen farms (Sweden) (Table 1). Thus, it is impossible to estimate the actual proportion of the year-round hunting season. No official data are available, but sometimes such hunting (against so-called pests) comes close to extermination. Moreover, our unpublished data show that in many regions of Europe, with developed hunting traditions, badgers are poached even if hunting is forbidden by law (Poland, Spain). Thus, in reality, the badger hunting bag and species extermination are higher than we could expect from direct calculations.

All the information presented above confirm that more attention should be paid to the situation/management of badgers, especially in large-area countries where long hunting seasons and high hunting bag coincide with other factors negatively affecting badger populations, e.g., roadkills. There are, therefore, several high-risk countries in Europe where long hunting seasons can significantly disturb badger populations. This is primarily the case in countries with hunting seasons longer than 3 months (Table 1).

## 5. Protection of Badger Setts

Badger setts play a crucial role in the ecology of the species, so some authors emphasized that special attention should also be paid to the protection of setts [52,133], where these animals spend much of their lives. Badger burrows have only been legally protected in the following European countries: Albania, Belgium, the Netherlands, United Kingdom, and Ireland [134]. Allowing badgers to be hunted with dogs during excavation and destruction of burrows is a serious threat. This is a widely accepted practice, e.g., in France and Sweden (Table 1). Another problem is observed in some areas of Spain, where it is allowed to hunt red foxes in burrows. As a result, it also generates a threat to badgers protected by law, which are killed “inadvertently” when their sett is confused with a red fox den. Therefore, setts/burrows should be protected even if a species is hunted, as in Polish hunting legislation, where all burrows are protected [135]. In Estonia, on the other hand, where badger is also a game species, large dens with more than ten entry holes are also protected [65]. Such a solution seems to be the right approach to badger conservation, especially when some authors report that badgers are sensitive to sett disturbance [136].

## 6. Trends

In Europe, badgers reach widely varying densities and numbers (Table 2). In southern Europe, the species is considered rare, e.g., in some regions of Spain [137]. In northern Italy, it was almost eradicated and thus species reintroduction has begun [138]. Reintroduction has also been attempted in the Netherlands [139]. The badger is not uncommon in Europe, although we cannot be sure of its population status as there are no reliable data. In fact, the low density of badgers in some regions may not only be the result of habitat capacity but simply the effect of intensive poaching [42].

Since the late 1990s [41,42], the hunting bag of this species has increased rapidly almost all over Europe ([150], Table 3). European countries can be divided into groups according to hunting bag size, which is a result of different areas and badger population densities. There are countries with a very high hunting bag increases, e.g., in Poland, almost 18-fold, but still with a relatively low yearly acquisition, not exceeding 10,000 individuals (also in Czechia, Slovakia, Estonia, and Switzerland). The situation seems to be different In Germanyy, where the hunting bag is also increasing, with a very high yearly acquisition (Table 3). In Germanyy, since the end of sett gassing, the annual hunting bag has increased from less than 5000 in the 1960s and 1970s to 79,900 individuals in 2018–19 [73,151].

However, there are many reports stating that badger populations are increasing in different parts of Europe (e.g., [161]), especially when hunting is suspended, but data on population trends expressed in numbers of individuals are scarce. Over a 9-fold increase in the badger population was observed in the Netherlands from 1983–2001 [139]. It should be noted that badgers have not been hunted in the Netherlands since 1994, and data sampling, started when there were almost no badgers in the environment. Between 1979 and 1995, an increase of 129% was reported in central Poland when hunting was curtailed [144]. An increase was also reported between 1992 and 2002, from nine to 27 families being placed under protection in Denmark [62]. Belgium also reported a 143% increase in badger populations (1988–1.15 ind/km^2^ and 1998–2.80 ind/km^2^), to a certain extent, as a result of full protection [162]. There is no information to confirm the real increase in population size basing on survey data. However, reports on hunting bag size alone seem to reveal a higher population increase than we could expect from the surveys. In general, hunting pressure is reducing badger populations rather than supporting their development [163].

The estimation of actual population trends based on game bag size can be misleading due to the possible impact of non-random variables, such as hunters’ preferences and tendencies, the increasing number of hunters, etc. [151]. However, such a practice of assessing population trends is common in many hunting societies in Europe. In fact, only sett surveys have given us the most reliable data on badger population trends, but such data are difficult to obtain and thus scarce. In western Europe, some authors reported an increase in population size after the extensive eradication by gassing of burrows in the 1960s and 1970s. In the Netherlands, badger populations started to grow when the species became protected by law [164]. The same situation applies to the following countries where the badger is protected: Luxembourg [86] and Spain [165]. There are some reports from regions that indicate an increase in population size [145,146] but under circumstances of suspended or limited hunting rather than its intensification. Excluding data based on the hunting bag, there is no report that reveals a strong population increase under hunting pressure.

Road mortality of badgers may provide some supplementary information on population trends, but there are usually no such data for longer periods, and in some cases, there has been no relationship between road accidents involving badgers and their population size [142]. Some authors report that, in general, the number of collisions with animals has increased in the last decades in Europe [166,167,168]. One of the reasons may be the increase in traffic, as well as animal population growth. In Switzerland, badger traffic casualties have increased by 113% between 1992 and 2015 [168], while the hunting bag has increased by 73% over a similar period (Table 3). Nevertheless, we still do not know whether the proportion between the hunting bag and the supposed population increase is correct. However, it seems that in some countries, these proportions are similar and distributed more reasonably. In western Poland, between 2010 and 2015, road mortality of badgers (excluding motorway opening effect) did not differ between years [169]. In relation to the data presented above, Poland belongs to a group of countries with very high hunting bag growth (Table 3). In line with country-dependent high hunting bag increases, there are no papers reporting an adequate increase of collisions with badgers greater than 100–200% in recent years [166,167,168,169]. Comparing these data with the rapidly and sharply increasing hunting bag, a question arises–is the badger population in some countries currently overhunted?

Hunting may constitute a significant share of the mortality of this species and cause its decline [12,145]. In the Carpathian Mountains in Poland, hunters obtain 0.37 badger/10 km^2^, while wolves kill 0.07 badger/10 km^2^ [43]. There are many indications that hunting negatively affects the badger population, as its size increases when hunting is limited [144]. Data from the Carpathian Mountains shows that as much as 65% of young badgers are killed annually as a result of hunting [43]. In northern Italy, the species has virtually disappeared as a result of intensive hunting management [138]. A similar situation had been reported in Denmark before the badger became a protected species [163].

## 7. Conclusions

Our great challenge is to halt the disappearance of species and habitats [170], so the role of the European badger in the ecosystem needs to be looked at with particular attention. More research needs to be done to consider whether the approach to a game animal can guarantee the maintenance of ecosystem services such as microhabitat creation, seed dispersal, and diversity shaping. It seems that in this matter, the importance of this mammal is still being underestimated. The real problem with appropriate badger management concerns countries that allow this species to be hunted in winter and spring during the mating season and parturition. Such a solution is supported in about a quarter of its European range. The real solution seems to be the reduction of the hunting season to two (October–November) or up to three months in late autumn (September–November). However, following solutions from other European countries, such as strict protection, should not be excluded either. Another problem concerns countries with a very high hunting bag increase in recent decades. In order to prevent overhunting in such circumstances, special attention should be paid to population monitoring.

## Figures and Tables

**Table 1:** Status of European badger *Meles meles* in Europe. Status: P = protected all year round; H = hunted; U = unclear: game species not hunted but also not protected. The date when the species was mentioned as fully protected is given in brackets. Updated in June 2020.

Country	Status P/H/U	Red List/Data Book	General Hunting Season	Supplementary Hunting Season and Remarks	Source
Albania	P [2008]	EN	–	Due to poor law enforcement and poaching, Albanian Government established in 2014 a hunting ban for two years in whole country. In 2016, that ban was extended till 2021.	[45,46]
Andorra	P [2013]	–	–	–	[47]
Austria	H	LC	Hunting season regulated only regionally.	01.04–31.03 Steiermark–all year; 01.06–01.01 Wien–in practice not hunted because of public security; 01.06–31.01 Burgenland, Kärnten; 16.06–31.01 Niederösterreich, 01.07–15.01 Oberösterreich; 01.07–28.02 Vorarlberg; 15.07–15.02 Tirol; 16.08–30.11 Salzburg.	[48,49,50]
Belarus	P [1981]	VU	–	–	[51]
Belgium	P [1992]	DD; VU	–	Hunting limited since 1973. VU status in Flanders and DD in Wallonia. In Brussels region very rare.	[52,53,54,55]
Bosnia and Herzegovina	H	–	All year	In Federation of Bosnia and Herzegovina, Republic of Srpska, and Brčko District, badger is not mentioned as protected species and hunting period is not defined thus in practice it can be hunted all year round.	[56,57]
Bulgaria	H	–	01.08–end of Feb	–	[58]
Croatia	H	–	01.08–31.12	–	[59]
Czechia	H	LC	01.09–30.11	Hunting permitted all year round where capercaillie, black grouse, hazel grouse and partridge occur.	[60,61]
Denmark	P [1994]	LC	–	–	[62,63]
Estonia	H	–	01.09–end of Feb	Setts with > 10 entrances protected by law.	[64,65]
Finland	H	–	01.08–31.03	Before 2014, an additional summer hunting season 01.05–31.07 was allowed (except females with cubs). Now not allowed.	[66]
France	H	LC	23.08/29.09–12.01/end of Feb	Departments: Bas-Rhin, Paris–hunting suspended. 15.05–…–supplementary period on may be announced and differed each year. Exact data of beginning and finishing of general hunting season published by prefects each year and depending on region vary between 23.08/29.09 to 12.01/end of Feb. Hunting with dogs, also burrow digging, is allowed.	[67,68,69]
Germany	H	–	01.08–31.10	All species occurring In Germanyy are mentioned in red list with proper category different from IUCN standards. In Germany red list, badger is considered as “ungefährdet” (not threatened). Berlin, Bremen–hunting suspended; 01.01–31.12 Mecklenburg–Vorpommern, Sachsen–all year; 01.01–31.12 Rheinland–Pfalz all year for juv., 01.08–31.12 for adults; 01.06–31.12 Baden–Württemberg for juv., 01.08–31.12 for adults; 01.08–31.10 Hessen, Saarland, Bayern; 01.08–15.01 Thüringen; 01.08–31.01 Brandenburg; Sachsen–Anhalt; Schleswig–Holstein, 01.09–30.11 Nordrhein–Westfalen; 01.09–31.01 Niedersachsen; 16.09–31.10 Hamburg.	[70,71,72,73]
Greece	U	NE	–	Populations from Crete and Rhodes are distinguished as subspecies of *M. canescens* (*M. c. arcalus* and *M. c. rhodius*).	[74]
Hungary	H	–	01.07–end of Feb	–	[75]
Ireland	P [1976]	LC	–	Licenses for badger cull have been granted in isolated areas to try to eradicate bovine tuberculosis.	[76,77,78]
Italy	U	LC	–	Italian Legge 157/92 (so-called game-law) lists all native species that can be hunted in Italy during hunting season. Badger is not listed, so it cannot be hunted.	[79,80]
Kosovo	H	LC	01.08–30.11	–	[81,82]
Latvia	H	–	01.08–31.03	–	[83]
Liechtenstein	H	–	01.09–31.12	–	[84]
Lithuania	H	–	01.10–01.12	–	[85]
Luxembourg	P [1986]	–	–	Hunting suspended since 1974. In 1986 badger became strictly protected by law.	[86,87]
Moldova	U	–	–	Game species but hunting suspended since 1995.	[88]
Montenegro	H	–	All year	Not estimated hunting season, so badger can be hunted all year round.	[89]
the Netherlands	P [1942]	–	–	Since 1942 hunting suspended, but in some cases hunting license was granted. As a result, hunting was not stopped until 1967, when badger became strictly protected with no exceptions.	[90,91]
North Macedonia	U	–	–	Mentioned as a game species but hunting suspended.	[92]
Norway	H	LC	21.08–31.01	–	[93,94]
Poland	H	–	01.09–30.11	Hunting permitted all year where capercaillie and black grouse occur.	[95]
Portugal	P [1986]	LC	–	–	[96,97]
Romania	H	–	01.08–31.03	–	[98]
San Marino	P [2007]	LC	–	–	[99,100]
Serbia	H	–	01.07–28.02	–	[101]
Slovakia	H	VU	01.09–30.11	–	[102,103]
Slovenia	H	–	01.08–31.12	–	[104]
Spain	P [1989]	LC	–	Categories of conservation status differ between autonomous regions, i.e., VU in Murcia. Real problems: predator control and hunting for foxes in burrows that may be inhabited by badgers.	[105,106,107]
Sweden	H	–	01.08–15.02	Hunting allowed all year round near human settlements, in gardens or when they pose a threat, on islands and near hen farms. 01.06–31.07–only for juveniles. Hunting with dogs, also in burrows, is allowed.	[108]
Switzerland	H	LC	16.06–15.01	Genève/Genf–hunting suspended; 16.06–15.01 Aargau, Basel–Stadt, Basel–Landschaft, Jura, Luzern, Nidwalden, St. Gallen, Schaffhausen, Solothurn, Thurgau, Ticino/Tessin, Zurich; 03.07–14.01 Appenzell, Ausserrhoden; 13.08–12.12 Neuchâtel/Neuenburg; 01.09-31.12 Bern; 01.09–15.01 Fribourg/Freiburg, Uri, Graubünden; 01.09–22.09 + 01.10–28(29).02 Schwyz; 01.09–24.09 + 07.10–15.01 Obwalden; 04.09–15.01 Appenzell, Innerrhoden; 17.09–15.01 Zug, Valais/Wallis; 01.10–15.01 Glarus; 01.01–15.01 + 01.10–31.10 Vaud/Waadt.	[109,110,111]
Turkey	P	–	–	–	[112]
Ukraine	H	–	01.10–30.11	–	[113]
United Kingdom	P	LC	–	Protected since 1985 in Northern Ireland, 1992 in England and Wales, 2011 in Scotland. Badgers are not hunted, with exceptions where it may be necessary to kill/harm/disturb them (a special license is needed). Each year, since 2013, UK government extends the number of areas with badger culling in order to eradicate bovine tuberculosis.	[114,115,116,117,118]

**Table 2:** Spatial characteristics of European badger *Meles meles* population in various parts of Europe. Status: P = protected all year round; H = hunted; U = unclear: game species not hunted but also not protected. The length of general hunting season given in brackets. For more detailed data see Table 1.

Region	Status/General Hunting Season	Population Density	Sett Density	Study Area	Source
		N/10 km^2^	Setts/10 km^2^	km^2^	
Scotland	P	22.0	–	1.21	[140]
British Isles	P	11.0–253.0	–	–	[141]
Wytham Woods, England	P	443.0	–	–	[142]
NE Poland	H (3)	5.9	1.0	–	[143]
C Poland	H (3)	3.1	0.3–1.1	–	[144]
Czechia	H (3)	0.6–7.1	–	–	[145]
N Moravia, Czechia	H (3)	1.2	4.51	–	[7]
NE Belarus	P	0.9	0.12	16–34	[123]
Białowieża Forest, Poland	H (3)	1.5–2.1	0.4–0.5	8–25	[39]
W Carpathians, Poland	H (3)	2.2	0.95	4.4–11.2	[125]
the Netherlands	P	4.0–22.0	–	0.8–1.1	[139]
Switzerland	H (7)	4.0–15.0	6.0	2.1–4.8	[146]
N Italy	U	9.3–14.0	3.4–5.1	0.9–3.2	[147]
S Spain	P	3.6	–	–	[148]
Portugal	P	3.6–4.8	–	–	[149]

**Table 3:** Trends in European badger *Meles meles* hunting bag (decrease in bold). Status: P = protected all year round; H = hunted; U = unclear: game species not hunted but also not protected. The length of general hunting season given in brackets. For more detailed data see Table 1.

Country	Status/General Hunting Season	Period	Number of Hunted Badgers		Trend of Acquisition		Source
			Start	End	%	↑↓	
Poland	H (3)	1990–2018	ca. 340	6400	+1782%	↑	[152]
Czechia	H (3)	1990–2018	ca. 490	4000	+720%	↑	[153,154]
Hungary	H (8)	2002–2018	1 649	12,394	+651%	↑	[155]
Estonia	H (6)	1992–2015	ca. 50	165	+230%	↑	[156]
Slovakia	H (3)	1990–2018	ca. 540	1395	+158%	↑	[157,158]
Germany	H (3)	1999–2019	33 824	79,900	+136%	↑	[159]
Switzerland	H (7)	1990–2018	1 812	3142	+73%	↑	[111]
Sweden	H (6.5)	1998–2018	30 408	23,593	−**22%**	↓	[160]

## Data Availability

Not applicable.
